# Ferroptosis in the U87MG Human Glioblastoma Cell Line Induces Damage Associated Molecular Phenotypes

**DOI:** 10.17912/micropub.biology.001524

**Published:** 2025-07-17

**Authors:** Leif Neitzel, Samantha Rea, Jessica Cornell, Charles Williams, Charles Hong

**Affiliations:** 1 Department of Medicine, Michigan State University College of Human Medicine, East Lansing, MI, USA; 2 Henry Ford Health + Michigan State Health Sciences, Detroit, MI, USA; 3 Department of Medicine, University of Maryland School of Medicine, Baltimore, Maryland, United States

## Abstract

Glioblastomas are known as “immune cold” cancers with little induction of damage-associated molecular phenotypes (DAMPS). We previously described the induction of ferroptosis in glioblastoma cells using the small molecule, OGM, a specific inhibitor of GPR68. The ferroptotic cell death pathway has been reported to induce the release of DAMPS. Here, we show that induction of ferroptosis through both Erastin and OGM results in DAMPS in U87MG cells. This suggests that ferroptosis in human glioblastomas may be able to convert them to an “immune hot” cancer, increasing their susceptibility to immunotherapy. These findings highlight the immunogenic potential of causing ferroptosis in glioblastoma as a therapeutic mechanism of action.

**Figure 1. Ferroptosis induces damage-associated molecular phenotypes in U87MG GBM cells:**
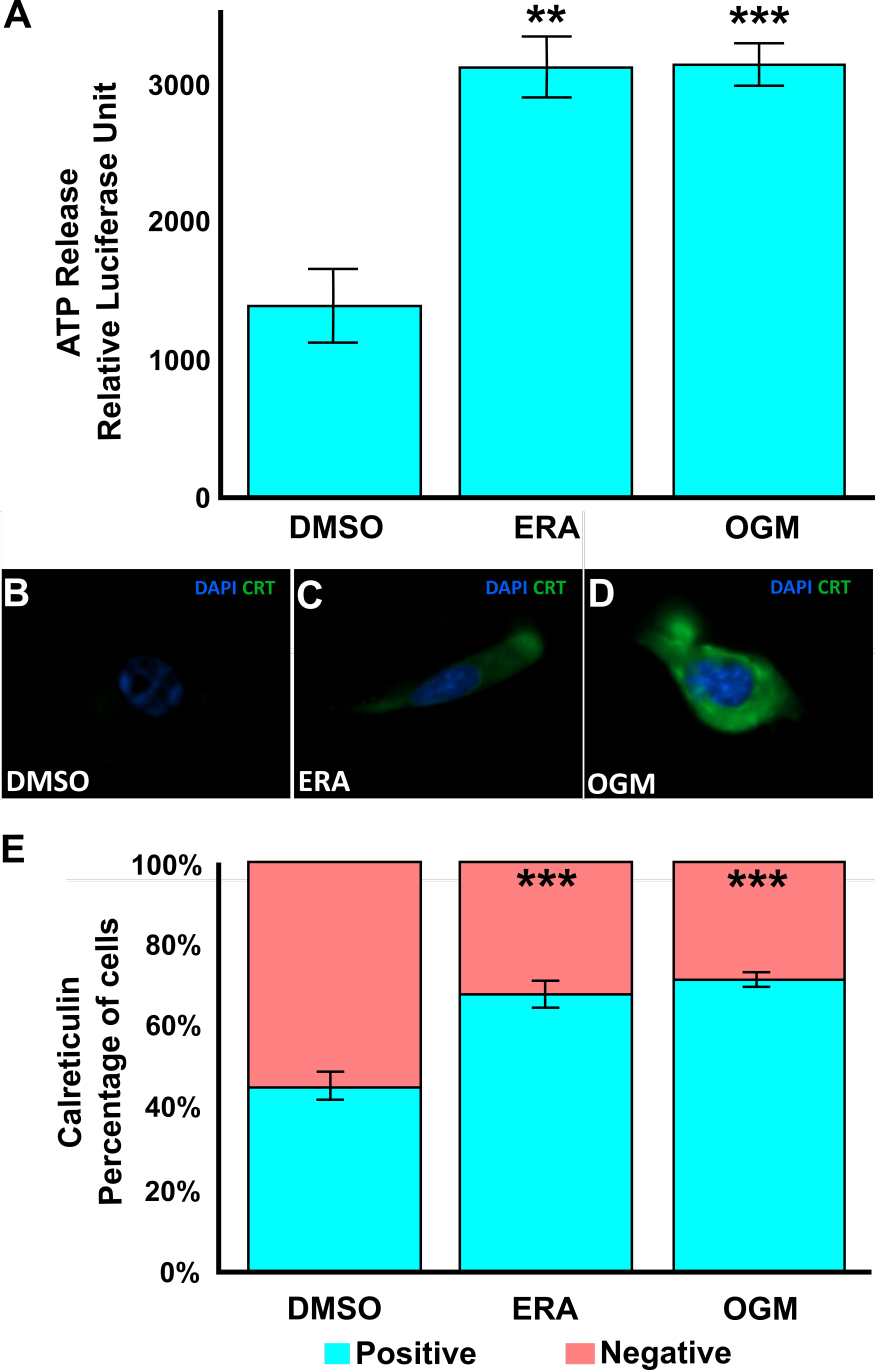
**(A)** Treatment of U87MG cells with ERA or OGM significantly increases ATP release into the media in comparison to the DMSO control. **(B)** , **(C)** , and ( **D)** Representative Immunofluorescence images of DAPI-stained nuclei (blue) and calreticulin staining (green) on the cell surface. ERA **(C)** and OGM **(D)** demonstrate a substantial increase in calreticulin on the cell surface in comparison to the DMSO-treated control **(B)** . **(E)** Quantification of the calreticulin-positive cells imaged in **(B)** , **(C)** , and ( **D),** mean % positive cells. **(A)** n=3 biological repeats with n=12 technical repeats. **(B)** , **(C)** , **(D)** , and **(E)** n=20 biological repeats with n≥200 cells per condition. Error bars show standard error of percent positive cells per well. **<p=0.005, ***<p=0.0005.

## Description

Glioblastoma multiforme (GBM) is the most prevalent malignant tumor of the central nervous system ^1–5^ . Immunotherapy represents a promising avenue for clinical cancer treatment, but thus far has been less efficacious in GBM due to the highly immune suppressive tumor microenvironment ^6–10^ . Ferroptosis, an iron-dependent form of regulated cell death, has garnered attention for its potential immunogenicity ^11–13^ . Ferroptosis is characterized by the release of damage-associated molecular patterns (DAMPs), which can trigger an immune response. Two key reporter DAMPs are adenosine triphosphate (ATP) release from the cells into the media and calreticulin (CRT) shuttling to the cell surface ^11–14^ .

While research has shown that ferroptosis can induce DAMPs in various cancer cell lines, direct evidence of this process specifically in GBMs is sparse ^12,15–22^ . Multiple studies have investigated the activation of ferroptosis in GBMs. However, these publications have not characterized the release of DAMPs, with the exception of a single paper using GL261 mouse glioma cells ^23^ . GL261 cells exhibited a significant initial increase in immunogenic potential that was gone by 24 hours of treatment ^23^ . Here, we will show, for the first time, direct evidence of DAMP release in a human glioblastoma cell line upon induction of ferroptosis. These findings are critical to understanding how ferroptosis might be harnessed therapeutically to stimulate anti-tumor immunity and improve treatment outcomes for GBM patients. Here we investigate ATP and calreticulin as readouts of immunogenic cell death (ICD) caused by the ferroptosis inducers Erastin (ERA), which inhibits the cystine-glutamate antiporter system Xc ^-^ , and Ogremorphin (OGM), a specific inhibitor of GPR68 ^21,24^ .

**Erastin and Ogremorphin cause ATP release and Calreticulin display in human GBM**

Treatment of the human glioblastoma multiforme cell line, U87MG, with ERA or OGM, resulted in a significant increase in ATP release, as measured by relative luminescence units (RLU) using the CellTiter-Glo assay. ERA induced a robust elevation in ATP secretion after 6 hours of treatment **(Figure A)** . Similarly, OGM resulted in a comparable enhancement in ATP release **(Figure A)** . Consistent with ATP release, treatment of U87MG cells with ERA and OGM also led to significant calreticulin exposure on the cell surface after 3 hours of treatment, a hallmark of immunogenic cell death (ICD) **(Figure B-D)** . Using immunofluorescence imaging and quantification both ERA and OGM induced a marked increase in calreticulin-positive cells **(Figure E)** . These results confirm that ferroptosis inducers like ERA and OGM can rapidly drive calreticulin shuttling and ATP release, key damage-associated molecular patterns (DAMPs). These data suggest a rapid increase in the immunogenic potential of GBMs undergoing early ferroptosis.

The ferroptosis inducers Erastin (ERA) and Ogremorphin (OGM) significantly enhance the immunogenicity of glioblastoma cells by driving the release of key damage-associated molecular patterns (DAMPs), including ATP and calreticulin exposure. These processes are critical for transforming an immune-cold tumor microenvironment into one capable of eliciting robust immune responses. In U87MG glioblastoma cells, treatment with ERA or OGM caused a significant elevation in ATP release, a critical marker of immunogenic cell death, effectively signaling immune activation in the extracellular environment. Additionally, calreticulin exposure, another hallmark of immunogenic cell death, was markedly increased within three hours of treatment, as observed through immunofluorescence imaging. These results underscore the potency and immediacy of the ferroptosis pathway in driving immunogenic cell death, providing compelling evidence for its role in converting immune-cold tumors into immune-hot tumors.

Together, these findings underline the dual role of ferroptosis in glioblastoma multiforme: early ferroptosis priming the tumor microenvironment for immune system activation while late ferroptosis simultaneously promotes iron-mediated cell death. By facilitating the release of ATP and the surface exposure of calreticulin, ferroptosis inducers like Erastin, which targets Xc-, and Ogremorphin, which targets GPR68, provide a promising avenue for enhancing the efficacy of immunotherapies in glioblastoma.

## Methods

**ATP experiments:**

U87MG cells were seeded in 12 well plates in DMEM (HEPES, high glucose, and GlutaMAX Supplement) and allowed to attach overnight at 37°C in 5% CO _2_ . Media was then removed and cells were washed with PBS before fresh FluoroBrite DMEM media containing 15 µM Erastin, 2 µM OGM, or control (DMSO) was added to the cells. Cells were then incubated at 37°C in 5% CO _2_ for 6 hours. The media was then removed and transferred to 96 well plates (20 µl per well) and ATP concentration in the media was quantified using CellTiter-Glo (100 µl per well). Luminescence was read with a Promega GloMax Multi.

**Calreticulin experiments:**

U87MG cells were seeded in a 96-well black-walled plates in DMEM (HEPES, high glucose, and GlutaMAX Supplement) and allowed to attach overnight at 37°C in 5% CO _2_ . The following day the media was replaced with fresh media containing 15 µM Erastin, 2 µM OGM, or control (DMSO). Cells were incubated for 3 hours at 37°C in 5% CO _2_ . Media was then removed and the cells were washed in PBS before being fixed with 4% PFA for 10 minutes at room temperature. Fixed cells were then washed with PBS and blocked in 5% donkey serum for 25 minutes at room temperature. After blocking, the 1° antibody (1:150, Calreticulin polyclonal) was added and samples were incubated at 4°C overnight. The following day, the wells were washed three times with PBS and the 2° antibody (1:200, Cy 2 conjugated AffiniPure Donkey Anti-Rabbit IgG) was added. Samples were incubated at room temperature for 2 hours. After incubation, cells were washed with PBS four times for 10 mins each. Cells were then mounted with Fluoroshield solution with DAPI and imaged on a Lionheart FX (Biotek-Agilent). Images were quantified using Gen5 (Biotek-Agilent).

**Statistical analysis**

Calreticulin increases with ICD, therefore a one-tailed Fisher’s exact test was used to determine significance of change in proportion of positive staining. Statistics on ATP release were preformed using multiple two-tailed student’s T-test. Both calculations used Bonferroni’s correction for multiple hypothesis testing.

## Reagents

**Reagents**

**Table:** 

**Reagent**	**Description**	**Vendor**	**Catalog Number**
**U87MG Cells**	Human glioblastoma cell line	ATCC	HTB-14
**Erastin (ERA)**	Ferroptosis inducer targeting Xc-	Sigma-Aldrich	SML1524
**Ogremorphin (OGM)**	GPR68 inhibitor and ferroptosis inducer	Custom Synthesized	N/A
**Cy2-conjugated Donkey Anti-Rabbit IgG**	Secondary antibody for calreticulin imaging	Jackson ImmunoResearch	711-225-152
**DAPI**	Nuclear stain for fluorescence microscopy	Sigma-Aldrich	F6057
**Cell Titer Glo Assay**	ATP quantification assay	Promega	G7571
**Fluoroshield with DAPI**	Mounting medium for fluorescence microscopy	Sigma-Aldrich	F6057
**DMEM, high glucose, GlutaMAX™ Supplement, HEPES**	Basal medium for maintaining U87MG cells	Gibco	10564011
**FluoroBrite DMEM**	DMEM with very low background fluorescence for imaging	Gibco	A1896701
**PBS, pH 7.4**	Washing cells	Gibco	10010023
